# Adaptive yoga versus low-impact exercise for adults with chronic acquired brain injury: a pilot randomized control trial protocol

**DOI:** 10.3389/fnhum.2023.1291094

**Published:** 2023-11-23

**Authors:** Jaclyn A. Stephens, Jesus A. Hernandez-Sarabia, Julia L. Sharp, Heather J. Leach, Christopher Bell, Michael L. Thomas, Agnieszka Burzynska, Jennifer A. Weaver, Arlene A. Schmid

**Affiliations:** ^1^Department of Occupational Therapy, Colorado State University, Fort Collins, CO, United States; ^2^Molecular Cellular and Integrative Neuroscience Program, Colorado State University, Fort Collins, CO, United States; ^3^Department of Health and Exercise Science, Colorado State University, Fort Collins, CO, United States; ^4^Sharp Analytics, LCC, Fort Collins, CO, United States; ^5^Department of Psychology, Colorado State University, Fort Collins, CO, United States; ^6^Department of Human Development and Family Studies, Colorado State University, Fort Collins, CO, United States; ^7^Center for Healthy Aging, Fort Collins, CO, United States

**Keywords:** traumatic brain injury, stroke, group exercise, heart rate variability, MRI, fMRI, fNIRS

## Abstract

**Background:**

Each year, millions of Americans sustain acquired brain injuries (ABI) which result in functional impairments, such as poor balance and autonomic nervous system (ANS) dysfunction. Although significant time and energy are dedicated to reducing functional impairment in acute phase of ABI, many individuals with chronic ABI have residual impairments that increase fall risk, decrease quality of life, and increase mortality. In previous work, we have found that yoga can improve balance in adults with chronic (i.e., ≥6 months post-injury) ABI. Moreover, yoga has been shown to improve ANS and brain function in healthy adults. Thus, adults with chronic ABI may show similar outcomes. This protocol details the methods used to examine the effects of a group yoga program, as compared to a group low-impact exercise, on primary and secondary outcomes in adults with chronic ABI.

**Methods:**

This study is a single-blind randomized controlled trial comparing group yoga to group low-impact exercise. Participants must be ≥18 years old with chronic ABI and moderate balance impairments. Group yoga and group exercise sessions occur twice a week for 1 h for 8 weeks. Sessions are led by trained adaptive exercise specialists. Primary outcomes are balance and ANS function. Secondary outcomes are brain function and structure, cognition, quality of life, and qualitative experiences. Data analysis for primary and most secondary outcomes will be completed with mixed effect statistical methods to evaluate the within-subject factor of time (i.e., pre vs. post intervention), the between-subject factor of group (yoga vs. low-impact exercise), and interaction effects. Deductive and inductive techniques will be used to analyze qualitative data.

**Discussion:**

Due to its accessibility and holistic nature, yoga has significant potential for improving balance and ANS function, along with other capacities, in adults with chronic ABI. Because there are also known benefits of exercise and group interaction, this study compares yoga to a similar, group exercise intervention to explore if yoga has a unique benefit for adults with chronic ABI.

**Clinical trial registration:**ClinicalTrials.gov, NCT05793827. Registered on March 31, 2023.

## 1 Introduction

Each year, an estimated 2.9 million Americans sustain acquired brain injuries (ABI) that result in emergency department visits, hospitalizations, and death (Schiller et al., 2012). ABI is an umbrella term that includes traumatic brain injuries (TBI) and non-traumatic injuries, including stroke, anoxic injuries, brain tumors, and other conditions that damage brain tissue. Although there are many treatment strategies in the early weeks and months after ABI, millions of individuals live with residual disability (Rutherford and Corrigan, 2009; Corrigan and Hammond, 2013; Whiteneck et al., 2016). This residual, chronic disability often includes significant impairments across multiple functional domains (e.g., motor, cognitive, emotional) and decreased brain function (Wilson et al., 2017). Two prominent and interconnected impairments – poor balance (McCulloch et al., 2010; Perez et al., 2018; Klima et al., 2019) and autonomic nervous system (ANS) dysfunction (Khalid et al., 2019; Xiong and Leung, 2019) – can together *and* independently increase fall risk, decrease quality of life, and increase mortality.

To date, there are few options for addressing impairments in adults with chronic ABI. However, community-based and holistic interventions, like hatha yoga, may be highly effective for simultaneously addressing balance (Green et al., 2019), ANS dysfunction (Zou et al., 2018) and may even improve brain function (Gothe et al., 2019). Hatha yoga includes breathwork, stretching, holding of postures, and meditation. Because stretching and holding postures requires *and* challenges balance, it is unsurprising that yoga interventions often elicit improvements in balance performance (Schmid et al., 2012; Stephens et al., 2020). Likewise, the breathwork and meditation components of hatha yoga may support improved ANS function, particularly for individuals with ABI. Briefly, ANS dysfunction following ABI is typically characterized by hyper-activation of the sympathetic nervous system, which can have damaging or even fatal impact on internal organs (Henden et al., 2014). It is theorized that the use of breathwork in hatha yoga may stabilize sympathetic and parasympathetic nervous system activity (Zou et al., 2018), but this has not yet been established for adults with ABI. Similarly, research with healthy adults has found that yoga can improve brain function (Gothe et al., 2019), but this has also not been tested in adults with chronic ABI. We theorize that yoga-induced improvements in functional abilities, like balance, are likely supported by improvements in brain structure and function.

Our research team has conducted two yoga feasibility studies with adults with chronic ABI to establish that our recruitment, retention, intervention, and assessment procedures (which includes neuroimaging methods) are feasible (Stephens et al., 2020, 2023). Notably, in these studies and other studies conducted by our team, we have seen that 8-weeks of yoga can improve balance and mobility (Stephens et al., 2020), cognition (Grieb et al., in press) and quality of life (Grimm et al., 2017). We have also have replicated that 8-weeks of yoga can improve balance in adults with chronic ABI, and we found preliminary evidence of improved brain function, even in the absence of measurable balance improvement (Stephens et al., 2023). Further, participants with chronic ABI have qualitatively shared that yoga improved a myriad of functional capacities (e.g., eating and swallowing, functional mobility, caring for pets, etc., Weaver et al., in press). Thus, our prior work suggests that yoga can support improvements across multiple functional domains. Still, the primary outcomes of interest for our current study are balance and ANS function, as they are the most logical areas of improvement given the components of hatha yoga (i.e., stretching/holding of postures and breathwork and meditation). Secondary outcomes include those that likely underpin functional improvements (i.e., changes in brain structure and function) and previously observed outcomes (i.e., cognition, quality of life, and qualitative experiences).

Specifically, our team is now conducting a single-blind pilot randomized control trial comparing two intervention arms: 8-weeks of group yoga and 8-weeks of low-impact group exercise in adults with chronic acquired brain injury. All prior studies (Grimm et al., 2017; Stephens et al., 2020, 2023) have been conducted as single-arm yoga intervention studies where participants served as their own controls, and baseline assessments were conducted before no-contact waiting periods and/or the intervention. Therefore, it is possible that the positive outcomes we have reported could be exclusively attributed to well-established benefits of physical exercise or social interaction. In order to determine if yoga has a distinctive impact on functional outcomes, like balance and ANS function, it is necessary to include a control group that mirrors many of the features of the yoga intervention, but lacks unique features of yoga (e.g., breath-to-movement connection). Thus, adding a low-impact group exercise control (Leach et al., 2023) is an essential next step to evaluate if yoga has a unique benefit for adults with chronic ABI. Again, our primary outcome measures are designed to evaluate balance performance and heart rate variability (HRV), as an indicator of ANS function. Secondary outcome measures are designed to: (1) measure neural underpinnings of functional improvements, specifically - functional and structural connectivity of neural networks and task-dependent neural activation during balance tasks and (2) evaluate previously observed outcomes of cognition, quality of life, and qualitative experiences. The overarching purpose of this manuscript is to describe, in detail, all components of our trial protocol to support transparency and replication of methods.

## 2 Methods and analysis

### 2.1 Study design and ethics

**Figure 1:**
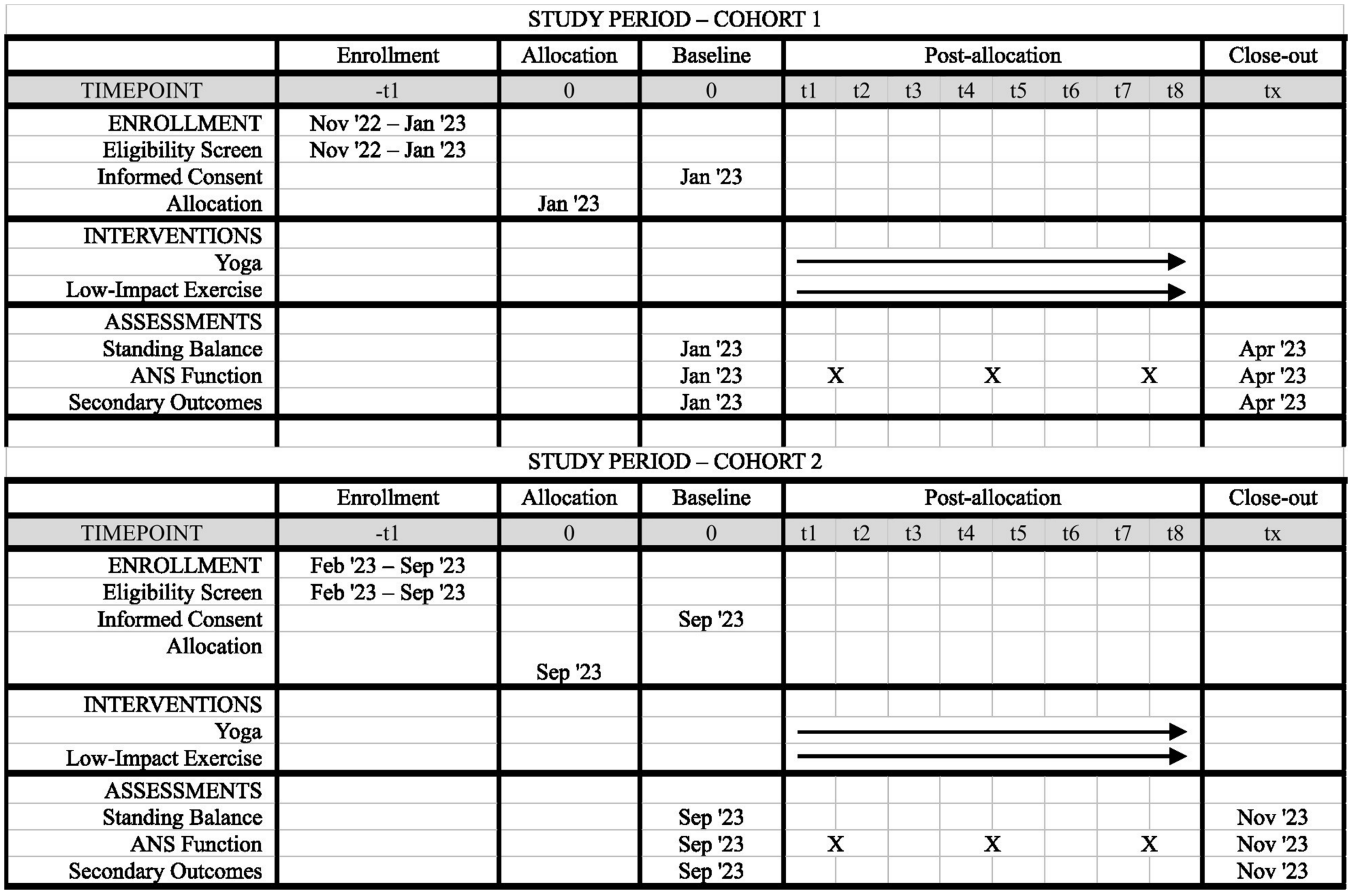
SPIRIT figure of study components. Timeline of enrollment, interventions, and assessments for participants in Cohort 1 and Cohort 2. The baseline time point is pre-intervention, and the close-out time point is post-intervention.

This study is being conducted within university laboratories and buildings for pre-and post-intervention assessments and interventions. All procedures are completed at a single site in the Mountain West subregion of the United States of America and described using SPIRIT reporting guidelines and checklist (Chan et al., 2013). The study consists of two participant cohorts: Cohort 1 and Cohort 2. A cohort design was selected to ensure small class sizes during yoga and control exercise sessions to support participant engagement, success, and safety. All study procedures for Cohort 1 and Cohort 2 are identical and the timeline of study components for both cohorts is depicted in Figure 1.

### 2.2 Selection of subjects

Participants are included if they are at least 18 years old, have sustained an ABI at least 6 months before start of study (operationally defined by our team as “chronic”), and have a self-reported balance impairment of moderate or greater, via the Neurobehavioral Symptom Inventory balance item (King et al., 2012). If completing magnetic resonance imaging (MRI) assessments, participants must meet standard screening criteria for a 3 Tesla scanner. To support successful recruitment, participants are not required to be yoga or exercise naïve, as the research was conducted in a relatively small community. Participants are excluded if they have not sustained an acquired brain injury or if they sustained an acquired brain injury within the past 6 months. They are also excluded if they self-report a mild balance impairment or no balance impairment, again via the neurobehavioral symptom inventory (King et al., 2012). The interventionists leading the yoga and low-impact exercise sessions were required to be a certified yoga instructor or personal trainer, respectively. Both interventionists had to be willing to follow a standardized protocol, designed by the co-principal investigators (Co-PIs), and complete human subjects training. Additionally, both interventionists were trained by occupational therapists on appropriate ways to communicate with and physically support individuals with brain injury during yoga and exercise.

### 2.3 Recruitment

Recruitment is ongoing and includes direct communication with local brain injury providers (e.g., physicians, occupational and physical therapists, etc.) and community organizations (e.g., Brain Injury Association of Colorado) to share information about the study via member list serves, quarterly newsletters, and support groups. Additionally, physical flyers have been posted and distributed within the local community and at the university, and radio and newspaper advertisements were used to expand reach.

### 2.4 Participant retention

To support participant retention, during eligibility screening, participants are fully informed of all study components and when each will occur. This is done to prevent loss of data and/or attrition due to known conflicts. Additionally, participants are sent reminders (via text message) for all study appointments, including pre-intervention assessment visits, yoga/exercise class sessions, and post-intervention assessment visits. To further support retention, attendance is taken at each class session, and participants are contacted if they miss a session and their reason for missing is recorded. Additionally, participant incentives are provided at the end of the last yoga/exercise class. Incentives are study materials – exercise mats, smart watches, and chest straps – that are used for each session and retained by the study team, so they are not lost or forgotten for sessions. Post-intervention assessment visits will be completed for all participants who participate in yoga/exercise sessions, but attendance data will be used to interpret post-intervention data, particularly for participants who miss multiple sessions.

### 2.5 Sample size

Power was determined by focusing on the primary outcome of balance. Thus, using balance data from our first feasibility study (Stephens et al., 2020), a power analysis was completed in G Power (version 3.118) (Faul et al., 2007) using a matched pairs t-test with a medium effect size (*d* = 0.5), a minimum power of 0.8 and a significance level of 0.05 to determine that 17 participants per group are needed (*n* = 34). We planned to enroll 48 participants due to potential attrition or enrollment challenges. Limited enrollment was achieved for Cohort 1 so planned enrollment is now 36 participants which still meets the minimum power to detect a medium effect size.

### 2.6 Random allocation of intervention arms

The study biostatistician created the allocation sequence. Up to twenty-four subjects for each cohort were randomly assigned to the yoga or exercise group. First, two blocks of 12 were created, and the randomization scheme was applied separately to each block. Within each block, 12 random numbers were generated using the pseudo-random number generating rand() function in Microsoft Excel. For the first cohort (where 12 individuals were recruited), six of those random numbers were associated with the yoga group and six were associated with the control group. The random number and associated group label were sorted from smallest random number to largest. The resulting randomization scheme was assigned to enrolled participants 001–012 such that six participants were randomly assigned to the yoga group and six participants were randomly assigned to the exercise group for the first cohort. The final allocation sequence was shared with the Co-PI and project manager, who is responsible for creating sealed envelopes that link participant IDs to group assignment. These steps will be repeated for the second cohort. Implementation of group assignment is completed in the following steps. First, the project manager or the PI screens participants for eligibility. Eligible participants are scheduled for a pre-intervention assessment visit where consenting, enrollment, and data acquisition are completed. Following consent, participants are given a unique participant ID number that is given in order of arrival to pre-intervention assessment visits. For example, the first person who arrives for a pre-intervention assessment is given a participant ID of 001. At the end of the pre-intervention visit, the project manager gives participants their sealed envelope, so they are aware of their group assignment.

### 2.7 Masking

For practical purposes, any research team member who provides or supports the interventions, including the Co-PI, are unmasked. Research team members who conduct pre-and post-intervention assessments, including the study PI, are masked to group assignment with one exception. Research team members who assess anatomy or physiology with instruments like MRI are not necessarily masked, as their knowledge of group membership is unlikely to influence outcomes. These individuals are not involved in data analysis where bias could be introduced. The study PI will only become unmasked in the instance of an adverse event, if the Co-PI is unable to share adequate details with the research compliance office (highly unlikely). Otherwise, the study PI will become unmasked after post-intervention assessments are completed and scored.

### 2.8 Interventions

Each intervention consists of 1-h sessions occurring twice per week for 8 weeks, for 16 total sessions. Interventions are delivered in classrooms within the same university building at the same time of day and day of the week. For this study, interventions are delivered in two cohorts. Specifically, up to 12 participants complete adaptive group yoga, and up to 12 participants complete low-impact group exercise in the first cohort, which occurs in the spring of 2023. Likewise, up to 12 participants complete adaptive group yoga, and 12 participants complete low-impact exercise in the second cohort, which occurs in the fall of 2023. In previous work, we have found that class sizes up to 12 participants is optimal for participant engagement and safety; thus, a cohort design was selected to reach enrollment numbers while ensuring participant safety. All study components, especially interventions, are delivered as similarly as possible from the same interventionists, so that participants from the two cohorts can be combined for analyses.

#### 2.8.1 Adaptive group yoga (active)

Hatha yoga is considered the foundation of yoga in the west and was used in both feasibility studies (Stephens et al., 2020, 2023). As in prior studies, yoga is delivered in a standardized progression using an established, manualized protocol. Sessions include breath work (pranayama), gentle stretching and holding of postures (asanas), mantra (repeated words), and meditation (dhyana); (see Stephens et al., 2023; Supplementary Table S1 for protocol details). Modifications are incorporated so all participants can successfully complete yoga sessions. During yoga sessions, the interventionist supports performance by modifying poses as appropriate, providing hands-on assistance to help participants move through poses, and using chairs/props to allow participants to complete postures.

#### 2.8.2 Low-impact group exercise (control)

The low-intensity exercise control group is matched to the estimated metabolic costs of yoga, 2.5 Metabolic Equivalent of Task (MET; Ainsworth et al., 2011). The exercise sessions were designed by members of the research team with significant experience in exercise protocol development (Leach et al., 2023), and included warm up, cool down, and five, 10-min exercise stations, (e.g., walking, balance, resistance bands, weight-bearing exercise, and core work); (see Leach et al., 2023 for protocol details). Exercise intensity was between 2.0 and 3.0 METs, equating to 30–40% heart rate reserve, and rating of perceived exertion (RPE) of 1–3, which represents light intensity (American College of Sports Medicine, 2017). Like the yoga intervention, exercise is delivered using an established manualized protocol. Heart rate zones are calculated for each participant and monitored along with RPE during exercise sessions to ensure fidelity to the prescribed intensity.

#### 2.8.3 Additional procedures for both interventions

All participants are informed that they can discontinue yoga or low-impact exercise at any time. In prior work (Stephens et al., 2020, 2022), there have been no adverse effects from yoga, but the research team is carefully monitoring participants at each yoga or exercise session for any adverse event, such as falls, fainting episodes, or significant discomfort. As noted, both interventions have manualized protocols to guide sessions to ensure adherence to the protocols. Additionally, adherence is being monitored for each yoga and exercise sessions with fidelity checklists. The Co-PI, who is not a masked assessor, reviews checklists throughout the intervention period, and – if necessary – meeting with interventionists to improve protocol adherence. Fidelity metrics are also being tracked for both interventions and will be reported with study outcomes. Finally, because participants are in a chronic phase of acquired brain injury recovery, they are rarely receiving concomitant care or other interventions. However, participants are asked to disclose concomitant care, interventions, or exercise protocols they might be following during the intervention period, but they are not prohibited from continuing with these activities.

### 2.9 Outcome measures

#### 2.9.1 Primary outcomes

Primary outcome data are acquired at pre-intervention and post-intervention assessments, and measures used to evaluate HRV are acquired throughout both interventions. Pre-intervention assessments are scheduled 2–3 weeks before start of yoga or exercise sessions, and post-intervention assessments are scheduled within 2 weeks of the last yoga or exercise session. See Figure 1 for additional detail on the participant timeline.

##### 2.9.1.1 Standing balance

Balance performance is assessed using two methods:

1. Balance is first assessed using a validated measure (Peller et al., 2023), the National Institutes of Health (NIH) Toolbox Standing Balance Task. This measure is administered using the NIH Toolbox app on an iPad (version 9) and the Balance Pod app on an iPhone (version 6), which are paired via Bluetooth. Prior to data acquisition, the study PI places a gait belt on the participant and secures the iPhone to the gait belt using a cellphone armband that is affixed to the gait belt. The Balance Pod app, via the iPhone, measures anterior–posterior sway during balance tasks which include: bipedal standing on a hard surface with eyes open; bipedal standing on a hard surface with eyes closed; bipedal standing on a soft surface (foam pad) with eyes open; bipedal standing on a soft surface with eyes closed; and tandem standing on a hard surface with eyes open. For all balance tasks, participants must remove their shoes and place their feet together with their heels and balls of feet touching. For eyes-open tasks, participants are instructed to fixate on an ‘X’ which is placed on wall in front of them at shoulder height. Tasks are administered in order of difficulty, starting with the easiest task (bipedal standing on a hard surface with eyes open). Each task lasts 50 s and can be repeated once if the participant has loss of balance (LOB) and steps out of the position. For safety purposes, participants complete all balance tasks in a corner, so they have walls on two sides if LOB occurs. The study PI also stands on their other side to stabilize participants in case of LOB. Only participants who make it to the fifth (and hardest task) complete the second balance assessment method.
2. Balance performance is also assessed using a previously validated (O'Connor et al., 2016; Goble et al., 2018; Richmond et al., 2018) force plate (Balance Tracking Systems, Inc., San Diego, CA) during simultaneous neuroimaging assessment with functional near-infrared spectroscopy (fNIRS). Four balance tasks are administered and include: bipedal standing on a hard surface with eyes open; bipedal standing on a hard surface with eyes closed; bipedal standing on a soft surface (foam pad) with eyes open; and bipedal standing on a soft surface with eyes closed. Each task is 30 s in duration, and tasks are repeated four times for a total of 16 trials, as task repetition is necessary to generate an averaged brain response. Balance performance for each trial is acquired with a sampling frequency of 25 Hz, and postural stability is quantified by measuring total center of pressure (COP) length, anterior–posterior COP length, and medio-lateral COP length. Again, for safety purposes, all balance trials are completed in a corner, and the study PI stands near the participant to stabilize them in instances of LOB. A masked study team member documents all instances of LOB during trials.

##### 2.9.1.2 Autonomic nervous system function

ANS function is also evaluated using two methods:

1. Prior to the start of the yoga and control exercise sessions, ANS function is evaluated using raw electrocardiogram (ECG) signals which are converted to HRV metrics (see statistical analysis section). Data are acquired at pre-intervention and post-intervention visits within a research lab and during yoga and control exercise sessions. In the research lab, study participants are situated on a medical bed, in a comfortable, semi-recumbent posture in a temperature-controlled (20–22 degrees Celsius), dimly lit room. During 11-min of metronome paced-breathing (6 breaths per minute) raw ECG signals, monitored via a physiological monitor (IntelliVue MP5 Patient Monitor, Philips Healthcare, United States), are recorded using a portable personal computer in conjunction with an analogue-to-digital convertor (WinDaq, Dataq Instruments Inc., Akron, Ohio, USA; Paxton et al., 2011; Williams et al., 2021).
2. During yoga and control exercise sessions, participants wear a Polar H9 chest strap and Unite watch^1^ to acquire and receive ECG signals; these signals are – like data acquired in the research lab – converted to HRV metrics offline after the raw ECG data are downloaded from the Polar cloud (footnote 1). Polar devices have been validated for HRV evaluation (Hernando et al., 2018) and considered the most accurate, commercially available HRV monitors (Stone et al., 2021).

ECG signals collected in the research lab and during yoga and exercise sessions via Polar devices are processed and analyzed offline using commercially available software (Kubios HRV; Tarvainen et al., 2014), as previously described (Tarvainen et al., 2002, 2014). The first 60-s are considered habituation while the study participant becomes accustomed to paced breathing; these data will be discarded from the final analysis.

#### 2.9.2 Secondary outcomes

Like primary outcomes, secondary and additional outcome data, with the exception of qualitative data (see below), are acquired at both pre-intervention and post-intervention assessment visits (see Figure 1).

##### 2.9.2.1 Functional connectivity and structural integrity of relevant neural networks

Imaging is performed on a 3 T MRI system (Siemens MAGNETOM Skyra) with a 64-channel radiofrequency (RF) coil. Anatomical scans are acquired using a 3D T1-weighted (T1w) magnetization prepared rapid gradient-echo (MP-RAGE) pulse sequence with the Generalized Autocalibrating Partially Parallel Acquisition (GRAPPA) enabled to reduce acquisition time [repetition time (TR) = 2,400 ms; TI = 1,000 ms; time to echo (TE) = 2.32 ms; flip angle = 8°; field of view (FOV) = 230 mm × 230 mm; matrix size = 255 × 255; in-plane resolution = 0.9 mm; slice thickness = 0.9 mm; slices = 192; slice spacing = 0; acceleration factor = 2; acquisition orientation = sagittal]. A fluid attenuated inversion recovery (FLAIR) sequence is used to screen for clinically significant white matter (WM) abnormalities [0.9 mm^2^, 192 slices, FOV read = 230 mm, TR = 5,000 ms, TE = 387 ms, inversion time (TI) = 1800 ms].

Next, to address specific study aims, the following three imaging methods are used to assess functional connectivity between select grey matter regions, and integrity of the relevant white matter tracts.

1. Resting state functional MRI (rs-fMRI): Functional scans sensitive to the T2-weighted blood-oxygen-level dependent (BOLD) signal are collected using a 2D gradient echo pulse sequence with interleaved acquisition [TR = 800 ms; TE = 38 ms; flip angle = 52°; FOV = 210 mm; matrix size = 84 × 84; in-plane resolution = 2.5 mm; slice thickness = 2.5 mm; slices = 54; slice spacing = 0; acquisition orientation = axial; interleaved, Siemens Simultaneous Multi-Slice (SMS) = 6 and Siemens integrated-Parallel Acquisition-Techniques (iPAT) = 2].

Detailed data preprocessing methods are provided in the supplement. Briefly, T1w and BOLD images are preprocessed using fMRIPrep 20.2.7 (Esteban et al., 2018a,b). T1w images are corrected for intensity non-uniformity (INU), skull-stripped, and then normalized to standard space (MNI152NLin2009cAsym). For each of the BOLD runs, a B0-nonuniformity map (or fieldmap) is estimated based on a phase-difference map calculated with a dual-echo GRE (gradient-recall echo) sequence used to correct for distortion. BOLD images are then co-registered to the T1w reference. Co-registration is configured with six degrees of freedom. Automatic removal of motion artifacts is completed using independent component analysis.

2. 3D-gradient and spin-echo (GRASE): Multi-echo T2 relaxation images are acquired in the transverse plane with a sequence (Prasloski et al., 2012). The duration of the GRASE sequence is 10.03 min (1.9 × 1.9 × 4 mm, 32 slices, distance factor 50%, TR = 1,000 ms, number of echoes = 32, first echo = 10 ms, echo spacing = 10 ms, flip angle = 90 degrees, FoV read = 240 mm).

3D GRASE images are fitted using an in-house script in Matlab (Prasloski et al., 2012). In brief, we use fitting of multiple exponential functions with stimulated echo correction using an extended phase graph to generate a T_2_ distribution from the multi-echo data. To improve the reliability of the fitting, we use Tikhonov regularization, which enforces minimum energy and generates a smooth T_2_ distribution. Finally, a myelin water fraction (MWF), which is the ratio of myelin water signal to total water signal, is calculated at each voxel. We used the integration ranges 10 ms – 40 ms for myelin water pool and 40 ms – 2000 ms for the axonal/extracellular water pool. Then, the first echo images from the 3D GRASE acquisition are linearly registered to the unwarped b = 0 diffusion-weight images (DWI) maps (6 degrees of freedom, 180 degrees search function, interpolation, sinc 15, see below), and this transformation is then applied to the individual MWF maps so that they are aligned with the b = 0 images, and, therefore, fractional anisotropy (FA) images.

3. Diffusion-weighted images (DWI) are acquired in the anterior-to-posterior phase encoding direction using a multi-shell (b = 0, 1,000, 2000) high-angular-resolution (138 directions) multiband echo planar sequence in transversal plane (1.8 mm3 voxels size, 76 slices, TR = 3,120, TE = 104.8, multiband acceleration factor = 4, flip angle = 79 degrees, refocus flip angle = 160 degrees). Then, the scan is repeated in the posterior-to-anterior phase encoding direction for one b = 0 image. This acquisition is designed to allow for fitting the Neurite orientation dispersion and density imaging (NODDI) model to the data, which combines a multi-shell high-angular-resolution diffusion imaging with three-compartment tissue modelling (intra-, extra-cellular and CSF; Le Bihan, 1995; Zhang and Meerschaert, 2011; Zhang et al., 2012). NODDI provides voxel-wise estimation of intra-cellular volume fraction (ICVF) reflecting axonal density, orientation dispersion index (ODI), and isotropic volume fraction (FISO; Zhang et al., 2012). Separate measurement of ICVF and FISO is critical for differentiating demyelination with and without axonal loss.

From the pairs of the DWIs acquired with reversed phase-encoding blips, susceptibility-induced off-resonance field is estimated (Andersson et al., 2003), as implemented in functional magnetic resonance imaging of the Brain Software Library (FSL) as top-up (Smith et al., 2004) generating a single corrected (or unwarped b = 0 image). Then, the top-up distortion correction is applied using eddy in FSL (Andersson and Sotiropoulos, 2016), which also corrects for distortions related to eddy current and motion. The diffusion tensor model can be fitted to the data using the FSL Diffusion Toolbox v.5.0 (FDT), yielding the FA maps. In addition, the NODDI model is fit to the data using NODDI toolbox in Matlab. All steps of image processing and registration are visually inspected. Finally, we use the tract-based spatial statistics (TBSS) (Smith et al., 2007) workflow in FSL v6.0.5.2. In brief, this includes: (a) nonlinear alignment of each participant’s FA volume to the 1 × 1 × 1 mm 3 standard Montreal Neurological Institute (MNI) space via the FMRIB58_FA template using the FMRIB’s Nonlinear Registration Tool (FNIRT, Rueckert et al., 1999),^2^ (b) calculation of the mean of all aligned FA images, (c) and the option to create a WM “skeleton” (a representation of WM tracts common to all subjects) by perpendicular non-maximum-suppression of the mean FA image and setting the FA threshold to 0.2, (d) perpendicular projection of the highest FA value (local center-of-tract) onto the skeleton, separately for each subject. Finally, we apply tbss_non_FA procedure to MWF and NODDI maps aligned with the unwarped b = 0 images, yielding MWF, FISO, and ICVF values projected on the skeleton.

##### 2.9.2.2 Region of interest analysis

To evaluate potential differences in specific WM tracts, we first estimate myelin content in the entire WM by averaging the MWF values from the entire skeleton for each participant (whole WM). Next, we extract mean MWF values from regions representing the major association, projection, and commissural fibers, as described previously (Burzynska et al., 2013), using the diffusion tensor imaging (DTI) white-matter atlas (Mori et al., 2005), with the aim to include the core regions specific to each tract. The corpus callosum is segmented so that genu represents prefrontal commissural fibers, body containing premotor, supplementary motor, motor and sensory fibers, and splenium containing parietal, temporal and occipital connections (Hofer and Frahm, 2006). The prefrontal WM region is defined as y > 12 in MNI coordinate space and whole WM included the whole TBSS skeleton. The cingulum includes both the ventral and dorsal segments, and the corticospinal tract is represented by the core regions of the superior corona radiata, posterior limb of the internal capsule, and the cerebral peduncles. The uncinate is probed in the lateral ventral prefrontal region as well as in the section along the fronto-occipital fasciculus. Mean MWF are extracted from each region using fslmeans in FSL.

##### 2.9.2.3 Task-dependent neural activation

These data are acquired via a single method using a portable fNIRS device, the NIRSport2 (nirx.net), during the second balance assessment method (see above for description).

FNIRS measures neural activation, via proxy measures of oxygenated hemoglobin (HbO) and deoxygenated hemoglobin (HbR), derived from superficial cortical regions. For this study, we are evaluating balance-task dependent activation in two ROI - bilateral inferior parietal and motor cortices, as these regions support postural control and balance (Surgent et al., 2019). To measure from these regions, we used the fNIRS Optodes’ Location Decider (fOLD) toolbox (Zimeo Morais et al., 2018) in Matlab to place thirty total optodes-15 light sources and 15 light detectors - over the ROIs in an fNIRS acquisition cap. Additionally, eight short separator detectors are included on the underside of the fNIRS cap to measure scalp perfusion, a nuisance variable, which is removed during data processing (Brigadoi and Cooper, 2015; Sato et al., 2016). FNIRS data are acquired via Aurora Version 2021.9 software (nirx.net).

Following acquisition, fNIRS data files are processed using Satori version 1.8.^3^ Preprocessing steps of conversion and spatial registration are performed automatically by Satori. Specifically, in the conversion step, the raw light intensity data are converted to optical density values and then converted to total hemoglobin (HbT), HbO and HbR values using the Modified Beer–Lambert Law (Baker et al., 2014). Then, all data are spatially registered to the fNIRS head probe and displayed for visual inspection. Following these automated steps, the data are segmented into ‘events,’ which represent the four balance tasks and their durations. Next, temporal pre-processing steps of motion artifact detection and correction are completed with a spike removal procedure that uses the Satori default parameters (10 iterations, 5 s lag, 3.5 threshold, 0.5 influence). A monotonic interpolation is applied in instances of spike detection. Next, to restore high frequency bands, the Temporal Derivative Distribution Repair (TDDR) (Fishburn et al., 2019) is used. To remove physiological noise, three steps are completed. First, a generalized linear model (GLM), which uses the highest correlation method to automatically select channels with artifacts, is completed for short separation regression (SSR). Next, low-frequency drifts as well as part of the non-hemodynamic related signal components such as heart rate are removed in a temporal filtering step using a Butterworth high-pass filter and then a Gaussian low-pass smoothing filter with cut-off frequencies of 0.01 Hz and 0.4 Hz, respectively. Finally, a normalization step using Z-transform is completed to make data comparable between subjects. Given the relativeness newness of Satori software, the default setting that automatically rejects channels with a scalp coupling index (SCI) below 0.75 (Pollonini et al., 2014) is not used. Instead, all data are processed without automated channel rejection (these are the data used group-level analyses), and then the raw data are processed again with the automated channel rejection step. This step generates a channel rejection map that indicates which channels would be rejected and their SCI value. This information is used to check data for potential outliers after group-level analysis. After all pre-processing was completed, group-level data can be generated using a multi-subject GLM approach within Satori.

##### 2.9.2.4 Cognition

Acquired via selected assessments from the NIH Toolbox Cognition Battery (Tulsky et al., 2014; Zelazo et al., 2014). The selected assessments include the List Sorting Working Memory Test which assesses working memory via tasks that require sequencing of visual and auditory stimuli, the Flanker Inhibitory Test which assesses inhibitory control and attention using a series of picture pairs, and the Dimensional Change Card Sort Test which assesses cognitive flexibility using target pictures. For each assessment, the NIH toolbox app converts raw scores to standardized scores for interpretation of performance. Specifically, for each assessment, raw scores are automatically converted into T-scores, along with an age-scored standard score (higher indicates better performance) and an age-adjusted national percentile (0–100%), which shows how the participant compares to age- and sex-matched peers (higher also indicates better performance).

##### 2.9.2.5 Self-reported quality of life

Acquired via the Traumatic Brain Injury Quality of Life (TBI-QOL) Short Form Measure on NIH Toolbox app (Tulsky et al., 2016). The NIH toolbox app converts raw scores to standardized scores for interpretation of performance. Specifically, raw scores are automatically converted into a metric, called T-scores. For short-form measures, the T-score Mean is 50, with a Standard Deviation of 10. T-scores above 50 indicate that individuals are having more problems in quality of life than age-and sex-matched individuals in the general population, whereas scores below 50 indicate that they are having fewer problems (Tulsky et al., 2016).

##### 2.9.2.6 Qualitative data

We will conduct qualitative focus groups and interviews with study participants to evaluate their experiences and perceptions of participating in an intervention study. These data are collected after the final session for both groups (i.e., post-intervention only).

#### 2.9.3 Additional outcome measures

##### 2.9.3.1 Body composition

Body composition, specifically body fat, is thought to be a determinant of autonomic nervous system activity (Poliakova et al., 2012; Triggiani et al., 2019). It is plausible that yoga may favorably modify body composition, and thereby introduce a potentially confounding variable. Accordingly, a physician’s digital scale, stadiometer, and dual energy x-ray absorptiometry (Hologic, Discovery W, QDR Series, Bedford, Massachusetts, United States) are used, as previously described (Ryan et al., 2020; Williams et al., 2021), to quantify changes in body mass, body mass index, and body composition.

### 2.10 Data collection

Primary and secondary outcome data are acquired during pre-intervention and post-intervention assessment visits, as outlined in earlier sections and Figure 1. To ensure quality of data acquisition, the following procedures were completed prior to participant enrollment. For NIH Toolbox data acquisition, the study PI and other assessors watched training videos, carefully reviewed the NIH Toolbox manual, and practiced administering the assessments. The study PI also trained assessors on fNIRS and force plate data acquisition, which included watching video tutorials, completing trial runs of procedures, and checking data quality of trial runs. For data acquisition related to evaluating ANS function, the study PI works closely with a co-investigator, who has expertise in HRV data acquisition and analysis (Williams et al., 2021); this co-investigator trained research team members who acquired pre-intervention and post-intervention ECG data. Additionally, the study PI communicated with Polar representatives and technical support personnel to confirm procedures for within-session data acquisition using Polar devices. Finally, study co-investigators have significant expertise in fMRI acquisition methods (Burzynska et al., 2013; Thomas et al., 2013, 2022; Mendez Colmenares et al., 2023) which supported appropriate selection of MRI sequences as outcomes; parameters for these sequences were also confirmed with an established MRI technician prior to any data acquisition.

### 2.11 Data management

All data are indexed using participant IDs to promote confidentiality, although the study PI has a list that links study ID to participant names. This list is on a password protected spreadsheet and saved to the PI’s personal network drive on a password protected computer. Raw data for all outcome measures are saved on university network drives, which are regularly backed-up by the IT department. Scored or summarized data are also stored within a Microsoft Access database which is managed by the study PI and the project manager. This database is also located on a university network drive. Most data can be imported into the Microsoft Access database directly from the acquisition program, which reduces the possibility of human error in data entry. Manual data entry is completed by a trained research assistant for participant demographic information including age, sex, race and ethnicity, type of ABI, time since injury, self-reported balance limitation, prescription medication use, and handedness. These data are reviewed by the study PI to ensure accuracy.

### 2.12 Data monitoring

The size of the study and associated budget did not support creation of a data monitoring committee. In addition to careful monitoring of possible adverse events during each yoga or exercise sessions, the study PI is responsible for evaluating all outcome data following post-intervention visits of the first cohort to assess if there are any significant safety concerns or poor outcomes that warrant termination of the trial.

#### 2.12.1 Harms

During each yoga or exercise session, interventionists are checking for and reporting any adverse events that happen before, during, or after the sessions. Interventionists have been provided a formal adverse events reporting form, which includes instructions on when to contact the Co-PI, PI, or – in extreme and unlikely circumstances – emergency services.

#### 2.12.2 Auditing

The size of the study and associated budget did not support creation of a formal auditing procedure. However, it is important to note that the funder is not involved in any aspect of data acquisition, interpretation, or dissemination of findings.

### 2.13 Statistical analysis of primary outcomes

#### 2.13.1 Balance data

T-Scores from the NIH Toolbox Standing Balance Task and center of pressure metrics from the force plate will use mixed effects statistical model to assess prospective within-subject (factor of time) and between-group (yoga vs. control) differences in balance performance.

#### 2.13.2 HRV data

There are multiple HRV metrics (e.g., time-series and frequency-band values) calculated within Kubios (Tarvainen et al., 2014). However, a review study showed consistent stabilization of the low frequency (LF) band power after yoga, reflecting an improved equilibrium of parasympathetic and sympathetic activity, (Zou et al., 2018) so initial analyses will use mixed effects statistical model to assess prospective within-subject (factor of time) and between group (yoga vs. control) differences in LF band power data.

### 2.14 Statistical analysis of secondary outcomes

#### 2.14.1 Functional connectivity data

This analysis plan will be guided by findings from behavioral data and completed in SPM 12, using false discovery rate (FDR) corrected contrasts to evaluate potential within and between-group differences, along with interaction effects, in intrinsic functional connectivity of specific neural networks (e.g., motor network).

#### 2.14.2 Structural integrity

This analysis plan will also be guided by findings from behavioral data using contrasts to evaluate within and between-group differences, along with interaction effects, in structural integrity of specific WM tracts.

#### 2.14.3 fNIRS data

HbO and HbR beta values will be used in a mixed effects model to assess prospective within and between-group differences, along with interaction effects, in neural activation over ROIs.

#### 2.14.4 Cognition

Standardized scores and T-scores from the List Sorting Working Memory Test, the Flanker Inhibitory Test, and the Dimensional Change Card Sort Test will be used in a mixed effects statistical model to assess prospective within and between-group differences, along with interaction effects, in cognition.

#### 2.14.5 Self-reported quality of life

Standardized scores and T-scores from the Traumatic Brain Injury Quality of Life Short Form Measure will be used in a mixed effects statistical model to assess prospective within and between-group differences, along with interaction effects, in self-reported quality of life.

#### 2.14.6 Qualitative data

Focus group and interview data are audio-recorded, transcribed verbatim, and de-identified. We will use NVivo software (QSR International Pty Ltd.) to support data analyses. We will use deductive and inductive analytic techniques. Deductively, we will analyze the transcripts for content related to the Consolidated framework for implementation research (CFIR) constructs (Damschroder et al., 2009), with intent to help explain elements of the intervention or study process that can be adapted for a future study. Inductively, we will use open, *in-vivo*, holistic coding (Glaser, 1965; Saldana, 2016). These open codes may be generated based on the participants’ perceptions of the intervention’s impact on their lives and to share how they perceived the intervention overall (e.g., related to ease of attendance, sense of engagement in sessions, etc.).

### 2.15 Additional statistical analysis plans for all outcomes

No additional statistical analyses of subgroups are planned at this time. Missing data will be managed with data removal; imputation is not planned.

## 3 Ethics and dissemination

### 3.1 Research ethics approval

All study procedures have been reviewed and approved by the Institutional Review Board (IRB) within Colorado State University’s Research Integrity and Compliance Review Office. Protocol approval number: 1799.

### 3.2 Protocol amendments

Initial IRB approval was provided in April 2021 for a feasibility study. Since then, the study PI has submitted a number of amendments to update study procedures and personnel to support completion of this pilot randomized control trial (RCT). Those procedures were approved in October 2022. The study PI will continue to inform the IRB of any changes to the study and request formal approval for any study amendments. If it highly unlikely that the PI will make any protocol changes that affect enrolled participants, however, if this occurs, participants will be informed of relevant study changes and re-consented, if necessary.

### 3.3 Consent

Written informed consent is acquired by the study PI and Co-PI from participants or their legal power of attorney. This study includes two consent forms (see appendices) – one for the main study and one specific to MRI procedures (this is only acquired from participants who are eligible for MRI). Consent is acquired during a pre-intervention assessment visit, which occurs after participants are screened for eligibility. To note, if participants are deemed ineligible for the study during screening, the research team retains none of their information, with the exception of their initial email correspondence for recruitment tracking. Written informed consent procedures include the following steps: (1) Participants are emailed consent form(s) in advance of their pre-intervention assessment visit. (2) Upon arrival at the pre-intervention assessment visit, participants review a printed copy of the consent form(s) with the PI or Co-PI, who clarify procedures and answer questions. (3) After questions are addressed, participants initial and sign consent form(s) and the PI or Co-PI also signs the form(s). All participants are offered a copy of the signed consent form at this time. (4) Throughout the study, participants are reminded that they can decline to complete any procedures, which has no penalty except possible removal from the study.

### 3.4 Confidentiality

Data confidentiality is maintained through steps outlined in the data management section. Additionally, participant confidentiality is maintained during research team member communication by only using initials or first names during electronic or verbal communication about participants, use of first names or initials on all study materials (e.g., polar watches include participant initials to avoid mix-ups), and use of secure communication when any sensitive information (e.g., participant phone numbers) are shared between team members.

### 3.5 Access to data

The study PI and Co-PI will have direct access to the complete final dataset. Other research team members will be granted access to the complete dataset if they submit a request with scientific merit to the study PI. Otherwise, research team members with certain expertise (e.g., with ANS data analysis) will likely only have access to those specific data to prevent accidental disruption, deletion, or corruption of data files.

### 3.6 Ancillary and post-trial care

Research team members are closely monitoring study outcomes (e.g., TBI-QOL measures) that may indicate participants need mental health support. The study team has prepared a list of local resources to share with participants in instances where mental health needs are identified. Provisions for compensation for those who suffer harm from trial participation are not available at this time, but they are unlikely to be needed, as prior studies have shown no adverse effects of yoga (and similar precautions are in place for exercise sessions).

### 3.7 Dissemination policy

The research team plans to disseminate study findings via peer-reviewed publications, conference abstracts, and within grant proposals. The study PI is also responsible for providing yearly updates to the Boettcher Webb-Waring Biomedical Research Award Foundation; this report will briefly describe the study status and group-level outcomes, once available. Authorship for peer-reviewed manuscripts and conference abstracts will be determined following the International Committee of Medical Journal Editors guidelines, and authorship will be discussed in initial stages of manuscript preparation. Finally, the research team will share yoga and exercise protocols upon request. The team also plans to share de-identified participant data in a public repository; however, this is pending acquisition of funds to provide training and time for submission by a team member.

## 4 Discussion

Individuals with chronic brain injury often experience poor balance and ANS dysfunction, which together can increase fall risk and reduce quality of life (McCulloch et al., 2010; Wilson et al., 2017; Perez et al., 2018; Khalid et al., 2019; Klima et al., 2019; Xiong and Leung, 2019). Here, we compare a group yoga intervention to a group low-impact exercise intervention and evaluate changes in balance and ANS function, along with the integrity of neural networks, task-dependent brain activity, cognition and quality of life. There is a growing body of literature on yoga for brain injury, but in prior work (Stephens et al., 2020), single-arm intervention designs were used. To determine if the positive outcomes observed after yoga are uniquely associated with yoga, this protocol introduces a comparable group exercise intervention in a pilot randomized control trial design. The benefits of exercise for acquired brain injury are well documented (An and Shaughnessy, 2011; van Duijnhoven et al., 2016) so we anticipate some degree of functional improvement in both groups. However, we also anticipate that the unique features of yoga may have a greater impact on certain functional outcomes.

### 4.1 Expected results

#### 4.1.1 Primary outcomes

Given our prior work (Schmid et al., 2012; Stephens et al., 2020), we expect significant improvements in balance as a result of the yoga intervention. However, we also expect that participants in the low-impact exercise group will experience improvements in balance. As such, we hypothesize significant within-subject differences in balance performance, but no between-group differences. We have different predictions for the ANS outcome. To-date, there is limited research investigating the effects of yoga on ANS function in populations with chronic acquired brain injury. Nevertheless, evidence from healthy adults indicate that yoga may improve ANS function (Zou et al., 2018). As such, we hypothesize that participants in the yoga group will have significantly greater improvements in ANS function than those in the low-impact experience group.

#### 4.1.2 Secondary outcomes

There is limited research on yoga on for brain injury, especially regarding its impact on the integrity neural networks and task-dependent brain activity. However, there is evidence from healthy populations and pilot data from our lab to inform our hypotheses. Specifically, in healthy individuals yoga can enhance structure and function of brain regions and neural networks, such as the default mode network (Gothe et al., 2019). Additionally, in adults with chronic acquired brain injury, pilot data from our lab suggests improved neural efficiency during balance tasks (Stephens et al., 2023). Based on these findings, we hypothesize that participants in the group yoga will have greater improvements in neural network integrity. We also expect to see decreased task-dependent brain activity in the yoga group, which would suggest improved neural efficiency after yoga.

Finally, we expect that yoga will have a significantly greater impact on cognitive function and quality of life. A recent meta-analysis showed yoga interventions improve cognition and quality of life in individuals with TBI (Acabchuk et al., 2021). Although this study did not include individuals with acquired brain injuries, we anticipate similar findings in our population.

In summary, we hypothesize that our protocol will elicit improvements in both groups, particularly on balance performance. However, given prior literature – mostly conducted with healthy populations – we predict that the unique features of yoga will have a significantly greater impact on ANS function, integrity of neural networks, task-dependent brain activity, cognition and quality of life.

## Ethics statement

The studies involving humans were approved by Colorado State University’s Research Integrity and Compliance Review Office. The studies were conducted in accordance with the local legislation and institutional requirements. The participants provided their written informed consent to participate in this study.

## Author contributions

JSt: Conceptualization, Data curation, Formal analysis, Funding acquisition, Investigation, Methodology, Project administration, Software, Supervision, Validation, Writing – original draft, Writing – review & editing. JH-S: Data curation, Formal analysis, Writing – review & editing. JSh: Conceptualization, Formal analysis, Methodology, Writing – review & editing. HL: Conceptualization, Methodology, Writing – review & editing. CB: Conceptualization, Formal analysis, Methodology, Writing – review & editing. MT: Conceptualization, Formal analysis, Methodology, Software, Writing – review & editing. AB: Conceptualization, Formal analysis, Methodology, Software, Writing – review & editing. JW: Writing – review & editing. AS: Conceptualization, Data curation, Funding acquisition, Investigation, Methodology, Project administration, Resources, Supervision, Validation, Writing – original draft, Writing – review & editing.

## Footnotes

^1^ polar.com

^2^ www.doc.ic.ac.uk/~dr/software

^3^ nirx.net/satori
