# Corrigendum: Adaptive yoga versus low-impact exercise for adults with chronic acquired brain injury: a pilot randomized control trial protocol

**DOI:** 10.3389/fnhum.2024.1369210

**Published:** 2024-02-13

**Authors:** Jaclyn A. Stephens, Jesus A. Hernandez-Sarabia, Julia L. Sharp, Heather J. Leach, Christopher Bell, Michael L. Thomas, Agnieszka Burzynska, Jennifer A. Weaver, Arlene A. Schmid

**Affiliations:** ^1^Department of Occupational Therapy, Colorado State University, Fort Collins, CO, United States; ^2^Molecular Cellular and Integrative Neuroscience Program, Colorado State University, Fort Collins, CO, United States; ^3^Department of Health and Exercise Science, Colorado State University, Fort Collins, CO, United States; ^4^Sharp Analytics, LCC, Fort Collins, CO, United States; ^5^Department of Psychology, Colorado State University, Fort Collins, CO, United States; ^6^Department of Human Development and Family Studies, Colorado State University, Fort Collins, CO, United States; ^7^Center for Healthy Aging, Fort Collins, CO, United States

**Keywords:** traumatic brain injury, stroke, group exercise, heart rate variability, MRI, fMRI, fNIRS

In the published article, an author name was incorrectly written as Agnieszka Z. Buryznska. The correct spelling is Agnieszka Burzynska.

The authors apologize for this error and state that this does not change the scientific conclusions of the article in any way. The original article has been updated.

