# Supplementary material for: Adaptive yoga versus low-impact exercise for adults with chronic acquired brain injury: a pilot randomized control trial protocol
Source: Front Hum Neurosci. 2023 Nov 23;17:1291094. doi: 10.3389/fnhum.2023.1291094 (PMC10701427; doi:10.3389/fnhum.2023.1291094)
Supplement: Supplementary file 1 [file Data_Sheet_1.DOCX]

Supplementary Material

# MRI Processing Methods

Note: These methods are autogenerated by fMRIprep.

T1w and BOLD images were preprocessed using fMRIPrep 20.2.7 (Esteban, Markiewicz, et al. (2018); Esteban, Blair, et al. (2018); RRID:SCR_016216), which is based on Nipype 1.7.0 (Gorgolewski et al. (2011); Gorgolewski et al. (2018); RRID:SCR_002502). T1w images were corrected for intensity non-uniformity (INU) with N4BiasFieldCorrection (Tustison et al. 2010), distributed with ANTs 2.3.3 (Avants et al. 2008, RRID:SCR_004757). The T1w-reference was then skull-stripped with a Nipype implementation of the antsBrainExtraction.sh workflow (from ANTs), using OASIS30ANTs as target template. Brain tissue segmentation of cerebrospinal fluid (CSF), white-matter (WM) and gray-matter (GM) was performed on the brain-extracted T1w using fast (FSL 5.0.9, RRID:SCR_002823, Zhang, Brady, and Smith 2001). A T1w-reference map was computed after registration of T1w image (after INU-correction) using mri_robust_template (FreeSurfer 6.0.1, Reuter, Rosas, and Fischl 2010). Brain surfaces were reconstructed using recon-all (FreeSurfer 6.0.1, RRID:SCR_001847, Dale, Fischl, and Sereno 1999), and the brain mask estimated previously was refined with a custom variation of the method to reconcile ANTs-derived and FreeSurfer-derived segmentations of the cortical gray-matter of Mindboggle (RRID:SCR_002438, Klein et al. 2017). Volume-based spatial normalization to two standard spaces (MNI152NLin2009cAsym, MNI152NLin6Asym) was performed through nonlinear registration with antsRegistration (ANTs 2.3.3), using brain-extracted versions of both T1w reference and the T1w template. The following templates were selected for spatial normalization: ICBM 152 Nonlinear Asymmetrical template version 2009c [Fonov et al. (2009), RRID:SCR_008796; TemplateFlow ID: MNI152NLin2009cAsym], FSL’s MNI ICBM 152 non-linear 6th Generation Asymmetric Average Brain Stereotaxic Registration Model [Evans et al. (2012), RRID:SCR_002823; TemplateFlow ID: MNI152NLin6Asym]. For each of the 6 BOLD runs the following preprocessing was performed. First, a reference volume and its skull-stripped version were generated using a custom methodology of fMRIPrep. A B0-nonuniformity map (or fieldmap) was estimated based on a phase-difference map calculated with a dual-echo GRE (gradient-recall echo) sequence, processed with a custom workflow of SDCFlows inspired by the epidewarp.fsl script and further improvements in HCP Pipelines (Glasser et al. 2013). The fieldmap was then co-registered to the target EPI (echo-planar imaging) reference run and converted to a displacements field map (amenable to registration tools such as ANTs) with FSL’s fugue and other SDCflows tools. Based on the estimated susceptibility distortion, a corrected EPI (echo-planar imaging) reference was calculated for a more accurate co-registration with the anatomical reference. The BOLD reference was then co-registered to the T1w reference using bbregister (FreeSurfer) which implements boundary-based registration (Greve and Fischl 2009). Co-registration was configured with six degrees of freedom. Head-motion parameters with respect to the BOLD reference (transformation matrices, and six corresponding rotation and translation parameters) are estimated before any spatiotemporal filtering using mcflirt (FSL 5.0.9, Jenkinson et al. 2002). The BOLD time-series (including slice-timing correction when applied) were resampled onto their original, native space by applying a single, composite transform to correct for head-motion and susceptibility distortions. These resampled BOLD time-series will be referred to as preprocessed BOLD in original space, or just preprocessed BOLD. The BOLD time-series were resampled into several standard spaces, correspondingly generating the following spatially-normalized, preprocessed BOLD runs: MNI152NLin2009cAsym, MNI152NLin6Asym. First, a reference volume and its skull-stripped version were generated using a custom methodology of fMRIPrep. Automatic removal of motion artifacts using independent component analysis (ICA-AROMA, Pruim et al. 2015) was performed on the preprocessed BOLD on MNI space time-series after removal of non-steady state volumes and spatial smoothing with an isotropic, Gaussian kernel of 6mm FWHM (full-width half-maximum). Corresponding “non-aggresively” denoised runs were produced after such smoothing. Additionally, the “aggressive” noise-regressors were collected and placed in the corresponding confounds file. Several confounding time-series were calculated based on the preprocessed BOLD: framewise displacement (FD), DVARS and three region-wise global signals. FD was computed using two formulations following Power (absolute sum of relative motions, Power et al. (2014)) and Jenkinson (relative root mean square displacement between affines, Jenkinson et al. (2002)). FD and DVARS are calculated for each functional run, both using their implementations in Nipype (following the definitions by Power et al. 2014). The three global signals are extracted within the CSF, the WM, and the whole-brain masks. Additionally, a set of physiological regressors were extracted to allow for component-based noise correction (CompCor, Behzadi et al. 2007). Principal components are estimated after high-pass filtering the preprocessed BOLD time-series (using a discrete cosine filter with 128s cut-off) for the two CompCor variants: temporal (tCompCor) and anatomical (aCompCor). tCompCor components are then calculated from the top 2% variable voxels within the brain mask. For aCompCor, three probabilistic masks (CSF, WM and combined CSF+WM) are generated in anatomical space. The implementation differs from that of Behzadi et al. in that instead of eroding the masks by 2 pixels on BOLD space, the aCompCor masks are subtracted a mask of pixels that likely contain a volume fraction of GM. This mask is obtained by dilating a GM mask extracted from the FreeSurfer’s aseg segmentation, and it ensures components are not extracted from voxels containing a minimal fraction of GM. Finally, these masks are resampled into BOLD space and binarized by thresholding at 0.99 (as in the original implementation). Components are also calculated separately within the WM and CSF masks. For each CompCor decomposition, the k components with the largest singular values are retained, such that the retained components’ time series are sufficient to explain 50 percent of variance across the nuisance mask (CSF, WM, combined, or temporal). The remaining components are dropped from consideration. The head-motion estimates calculated in the correction step were also placed within the corresponding confounds file. The confound time series derived from head motion estimates and global signals were expanded with the inclusion of temporal derivatives and quadratic terms for each (Satterthwaite et al. 2013). Frames that exceeded a threshold of 0.5 mm FD or 1.5 standardized DVARS were annotated as motion outliers. All resamplings can be performed with a single interpolation step by composing all the pertinent transformations (i.e. head-motion transform matrices, susceptibility distortion correction when available, and co-registrations to anatomical and output spaces). Gridded (volumetric) resamplings were performed using antsApplyTransforms (ANTs), configured with Lanczos interpolation to minimize the smoothing effects of other kernels (Lanczos 1964). Non-gridded (surface) resamplings were performed using mri_vol2surf (FreeSurfer).

Abraham, Alexandre, Fabian Pedregosa, Michael Eickenberg, Philippe Gervais, Andreas Mueller, Jean Kossaifi, Alexandre Gramfort, Bertrand Thirion, and Gael Varoquaux. 2014. “Machine Learning for Neuroimaging with Scikit-Learn.” Frontiers in Neuroinformatics 8. https://doi.org/10.3389/fninf.2014.00014.

Avants, B.B., C.L. Epstein, M. Grossman, and J.C. Gee. 2008. “Symmetric Diffeomorphic Image Registration with Cross-Correlation: Evaluating Automated Labeling of Elderly and Neurodegenerative Brain.” Medical Image Analysis 12 (1): 26–41. https://doi.org/10.1016/j.media.2007.06.004.

Behzadi, Yashar, Khaled Restom, Joy Liau, and Thomas T. Liu. 2007. “A Component Based Noise Correction Method (CompCor) for BOLD and Perfusion Based fMRI.” NeuroImage 37 (1): 90–101. https://doi.org/10.1016/j.neuroimage.2007.04.042.

Dale, Anders M., Bruce Fischl, and Martin I. Sereno. 1999. “Cortical Surface-Based Analysis: I. Segmentation and Surface Reconstruction.” NeuroImage 9 (2): 179–94. https://doi.org/10.1006/nimg.1998.0395.

Esteban, Oscar, Ross Blair, Christopher J. Markiewicz, Shoshana L. Berleant, Craig Moodie, Feilong Ma, Ayse Ilkay Isik, et al. 2018. “FMRIPrep.” Software. Zenodo. https://doi.org/10.5281/zenodo.852659.

Esteban, Oscar, Christopher Markiewicz, Ross W Blair, Craig Moodie, Ayse Ilkay Isik, Asier Erramuzpe Aliaga, James Kent, et al. 2018. “fMRIPrep: A Robust Preprocessing Pipeline for Functional MRI.” Nature Methods. https://doi.org/10.1038/s41592-018-0235-4.

Evans, AC, AL Janke, DL Collins, and S Baillet. 2012. “Brain Templates and Atlases.” NeuroImage 62 (2): 911–22. https://doi.org/10.1016/j.neuroimage.2012.01.024.

Fonov, VS, AC Evans, RC McKinstry, CR Almli, and DL Collins. 2009. “Unbiased Nonlinear Average Age-Appropriate Brain Templates from Birth to Adulthood.” NeuroImage 47, Supplement 1: S102. https://doi.org/10.1016/S1053-8119(09)70884-5.

Glasser, Matthew F., Stamatios N. Sotiropoulos, J. Anthony Wilson, Timothy S. Coalson, Bruce Fischl, Jesper L. Andersson, Junqian Xu, et al. 2013. “The Minimal Preprocessing Pipelines for the Human Connectome Project.” NeuroImage, Mapping the connectome, 80: 105–24. https://doi.org/10.1016/j.neuroimage.2013.04.127.

Gorgolewski, K., C. D. Burns, C. Madison, D. Clark, Y. O. Halchenko, M. L. Waskom, and S. Ghosh. 2011. “Nipype: A Flexible, Lightweight and Extensible Neuroimaging Data Processing Framework in Python.” Frontiers in Neuroinformatics 5: 13. https://doi.org/10.3389/fninf.2011.00013.

Gorgolewski, Krzysztof J., Oscar Esteban, Christopher J. Markiewicz, Erik Ziegler, David Gage Ellis, Michael Philipp Notter, Dorota Jarecka, et al. 2018. “Nipype.” Software. Zenodo. https://doi.org/10.5281/zenodo.596855.

Greve, Douglas N, and Bruce Fischl. 2009. “Accurate and Robust Brain Image Alignment Using Boundary-Based Registration.” NeuroImage 48 (1): 63–72. https://doi.org/10.1016/j.neuroimage.2009.06.060.

Jenkinson, Mark, Peter Bannister, Michael Brady, and Stephen Smith. 2002. “Improved Optimization for the Robust and Accurate Linear Registration and Motion Correction of Brain Images.” NeuroImage 17 (2): 825–41. https://doi.org/10.1006/nimg.2002.1132.

Klein, Arno, Satrajit S. Ghosh, Forrest S. Bao, Joachim Giard, Yrjö Häme, Eliezer Stavsky, Noah Lee, et al. 2017. “Mindboggling Morphometry of Human Brains.” PLOS Computational Biology 13 (2): e1005350. https://doi.org/10.1371/journal.pcbi.1005350.

Lanczos, C. 1964. “Evaluation of Noisy Data.” Journal of the Society for Industrial and Applied Mathematics Series B Numerical Analysis 1 (1): 76–85. https://doi.org/10.1137/0701007.

Power, Jonathan D., Anish Mitra, Timothy O. Laumann, Abraham Z. Snyder, Bradley L. Schlaggar, and Steven E. Petersen. 2014. “Methods to Detect, Characterize, and Remove Motion Artifact in Resting State fMRI.” NeuroImage 84 (Supplement C): 320–41. https://doi.org/10.1016/j.neuroimage.2013.08.048.

Pruim, Raimon H. R., Maarten Mennes, Daan van Rooij, Alberto Llera, Jan K. Buitelaar, and Christian F. Beckmann. 2015. “ICA-AROMA: A Robust ICA-Based Strategy for Removing Motion Artifacts from fMRI Data.” NeuroImage 112 (Supplement C): 267–77. https://doi.org/10.1016/j.neuroimage.2015.02.064.

Reuter, Martin, Herminia Diana Rosas, and Bruce Fischl. 2010. “Highly Accurate Inverse Consistent Registration: A Robust Approach.” NeuroImage 53 (4): 1181–96. https://doi.org/10.1016/j.neuroimage.2010.07.020.

Satterthwaite, Theodore D., Mark A. Elliott, Raphael T. Gerraty, Kosha Ruparel, James Loughead, Monica E. Calkins, Simon B. Eickhoff, et al. 2013. “An improved framework for confound regression and filtering for control of motion artifact in the preprocessing of resting-state functional connectivity data.” NeuroImage 64 (1): 240–56. https://doi.org/10.1016/j.neuroimage.2012.08.052.

Tustison, N. J., B. B. Avants, P. A. Cook, Y. Zheng, A. Egan, P. A. Yushkevich, and J. C. Gee. 2010. “N4ITK: Improved N3 Bias Correction.” IEEE Transactions on Medical Imaging 29 (6): 1310–20. https://doi.org/10.1109/TMI.2010.2046908.

Zhang, Y., M. Brady, and S. Smith. 2001. “Segmentation of Brain MR Images Through a Hidden Markov Random Field Model and the Expectation-Maximization Algorithm.” IEEE Transactions on Medical Imaging 20 (1): 45–57. <https://doi.org/10.1109/42.906424>.

# Main Study Consent Form

**Consent to Participate in a Research Study**

**Colorado State University**

**Title of Study:** Neural Markers of Static & Dynamic Balance in Adults with Acquired Brain Injury

**Principal Investigators:**

Jaclyn Stephens, PhD, Dept. of Occupational Therapy

[jaclyn.stephens@colostate.edu](mailto:jaclyn.stephens@colostate.edu)

Arlene Schmid, PhD, Dept. of Occupational Therapy

[arlene.schmid@colostate.edu](mailto:arlene.schmid@colostate.edu)

Jennifer Weaver, PhD, Dept. of Occupational Therapy

[jen.weaver@colostate.edu](mailto:jen.weaver@colostate.edu)

Christopher Bell, PhD, Dept. of Health and Human Sciences

[christopher.bell@colostate.edu](mailto:christopher.bell@colostate.edu)

**Why am I being invited to take part in this research?**

You have responded to our study advertisement, you are an adult who had an acquired brain injury or stroke more than 6 months ago, and you are interested in being a volunteer in our research study.

**Who is doing the study?**

Faculty members (listed above) from the Department of Occupational Therapy and Health and Human Sciences are conducting this study. Graduate and undergraduate students who work with these faculty members will also be involved in this research.

**What is the purpose of this study?**

The purpose of the research is to see if group yoga or group exercise can influence brain activity, balance performance, physiological functions, like heart rate, or other capacities, like memory. By measuring your brain activity with functional near-infrared spectroscopy (fNIRS) while you are performing balance tasks, we can learn if brain activity and balance abilities change after group yoga or group exercise. By completing body composition scans and measuring heart rate, we can learn if your physiological functions change after group yoga or group exercise. Finally, by using self-report measures that ask about your capacities, like memory, we can see if these change after group yoga or group exercise.

**Where is the study going to take place and how long will it last?**

This study will take place in three locations on the Colorado State University (CSU) campus: at the Translational Medicine Institute (TMI), the Human Performance Clinical Research Laboratory (HPCRL), and the Center for Healthy Aging at CSU’s Health and Medical Center. We ask that you participate in a pre-intervention assessment (at TMI and HPCRL), sixteen sessions of group yoga or group exercise (at the Center for Healthy Aging), and a post-intervention assessment (at TMI and HPCRL). Each assessment will last 2-2.5 hours, and the group yoga and group exercise classes will last 1 hour, twice a week, for 8 weeks. We will also invite you to complete a post-intervention interview with us, which will be take 30-60 minutes and will be completed online. Thus, the total time commitment will be up to 22 hours. The assessment visits and post-intervention interview will be scheduled to fit easily into your schedule.

**What will I be asked to do?**

You will be asked to complete the following activities:

Demographic Questionnaire: You will be asked to fill out a questionnaire about personal information, such as sex, age, race, and if you have or have had any neurological or psychiatric conditions so that we can determine if certain factors influence brain activity during balance tasks or influence your physiological functions. This questionnaire will take you between 5-10 minutes to complete.

Self-Report and Brief Performance Measures: You will be asked to answer questions about your general health, fall history, experience with yoga, pain, balance, mental health, mental abilities (like memory, thinking, planning, and organizing) and quality of life. You will also be asked to complete a couple brief performance measures that assess your mental and physical abilities (like memory, planning, reaction time, and standing balance). These measures will take you between 20-30 minutes to complete.

fNIRS: A cap, similar to a bathing cap, with functional near infrared spectroscopy (fNIRS) optodes will be placed on your head. The optodes in the cap will emit light and detect refracted light to measure your brain activity by evaluating blood oxygen levels. It will take between 10 to 15 minutes to place the cap on your head and set up equipment. It will take between 20 to 30 minutes to complete balance tasks while wearing the fNIRS cap, and it will take 1-2 minutes to remove the cap. While you are wearing the cap, you will be asked to complete balance tasks, and we will videotape your performance so we can rate it after you leave. This video recording will only be used by the research team for training purposes.

Body Composition: It is possible that over the 8-weeks of group yoga or exercise, the amounts of muscle and fat you have might change. We will measure how much fat and muscle you have in your body using a test called dual energy x-ray absorptiometry (DEXA). The DEXA test requires you to lie quietly on a padded table while a small probe gives off low-level x-rays and sends them over your entire body. This test gives very accurate measurements of your body fat and bone mineral density and takes less than 15 minutes to complete.

Heart Rate: It is also possible that over the 8-weeks of study participation, your resting heart rate might change. After each of the measurements of body composition we will ask you to lie on a bed for 12-minutes while we measure your heart rate. During the measurement we will ask you to breathe in time to a metronome. Your breathing rate will be 12 breaths per minute. You will be asked to not eat of drink anything (including coffee) for 8-hours before this measurement. Drinking water will be allowed. You will also be asked not to perform difficult exercise during the 12-hours prior to this measurement.

Group Yoga OR Group Exercise: You will be assigned to complete either group yoga or group exercise; you will not have a choice of group. Both group yoga and group exercise classes will last 1 hour per class, with classes twice a week, for 8 weeks (i.e. 16 total classes). These classes will be led by trained instructors, and class activities can be modified to support your participation.

Post-Intervention Interview: Approximately 2-3 weeks after group yoga or group exercise, you will be asked to meet with a member of the research team via a video conferencing platform, like Zoom. The researcher will ask you questions about your experience in the study and record your responses. This interview will last 30-60 minutes and will be recorded.

- Checking this box indicates that you consent to your interview being recorded. If you do not consent to having your interview recorded, you will not participate in the interview.

You may be asked to do:

Magnetic Resonance Imaging (MRI): If you take part in this, you will complete a separate consent form that thoroughly describes our MRI procedures and acquires additional consent.

**Are there reasons why I should not take part in this study?**

If you currently have or have history of neurological conditions other than brain injury (e.g., epilepsy) or psychiatric conditions (e.g., schizophrenia or bipolar) you will not be able to participate in this study. If you are pregnant, you should not participate in the body composition scan.

**What are the possible risks and discomforts?**

FNIRS: The fNIRS recordings are performed according to standard practices within the field. There are no known risks in recording your brain activity with fNIRS. In placing scalp or other skin-surface sensors or optodes it is possible that you may experience slight tenderness in those locations, especially if you have very sensitive skin. This tenderness should disappear quickly. Please inform us if you have a history of fainting in the doctor’s office or other location when excited. It is not possible to identify all potential risks in research procedures, but the researcher(s) have taken reasonable safeguards to minimize any known and potential, but unknown, risks.

Body Composition (DEXA) Scan: The risks associated with the DEXA are very low. The maximum radiation dose you will receive in this study is less than 1/1000th of the federal and state occupational whole-body dose limit allowed to radiation workers (5,000 mrem). Put another way, the maximum dose from any scan we utilize with this DEXA ranges from 1.2 mrem (whole-body scan) to 12.2 mrem (for several of the regional scans, such as lumbar, femur, and forearm scans). The average annual background radiation you already receive is at least 620 mrem/year. The more radiation you receive over the course of your life, the more the risk increases of developing a fatal cancer or inducing changes in genes. The radiation in this scan is not expected to significantly increase these risks, but the exact increase in such risks is not known. There are no discomforts associated with this procedure. Women who are or could be pregnant should receive no unnecessary radiation and should not participate in this study.

Resting Heart Rate: There are no known risks of lying on a bed for 12-minutes while having your heart rate measured. Skipping breakfast (i.e. not eating for 8-hours) may make you might feel hungry.

**Are there any benefits from taking part in this study?**

There are no direct benefits for participants. However, we find that participants typically are interested in seeing their brain activity displayed on the screen. The results from this research will help us understand if group yoga or group exercise can influence brain activity, balance performance, physiological functions, like heart rate, or other capacities, like memory

**Do I have to take part in the study?**

Your participation in this research is voluntary. If you decide to participate in the study, you may withdraw your consent and stop participating at any time without penalty or loss of benefits to which you are otherwise entitled.

**Who will see the information that I give?**

We will keep private all research records that identify you, to the extent allowed by law. All information collected for this study will be only identified by a participant code and not by your name or any other personal information in the computer or written forms. An example of the code that will be used is S002 (S=study and 002=the participant number). The list linking your name to the code used for your information will be kept in a computer file that requires a password to access and only the research team will have access to that list. We will destroy the list once we have published the research information. We will make every effort to prevent anyone who is not on the research team from knowing that you gave us information, or what that information is. For example, your consent form that has your name will be kept separate from other research records about you and these two things will be stored in different places under lock and key. We may publish the results of this study; however, we will keep your name and other identifying information private. Your information will be combined with information from other people taking part in the study. You will not be identified in these written materials.

**Can my taking part in the study end early?**

If you are unable to participate in multiple group yoga or group exercise classes, or if you don’t want to wear the cap for the fNIRS portions of this study, you may be removed from the study.

**Will I receive any compensation for taking part in this study?**

All participants will receive compensation of an exercise mat, a chest strap device, and a smart watch. These items are valued around $250.

**What if I have questions?**

Before you decide whether to accept this invitation to take part in the study, please ask any questions that might come to mind now. Later, if you have questions about the study, you can contact the investigators, Jaclyn Stephens at [jaclyn.stephens@colostate.edu](mailto:jaclyn.stephens@colostate.edu) or Arlene Schmid at [arlene.schmid@colostate.edu](mailto:arlene.schmid@colostate.edu). If you have any questions about your rights as a volunteer in this research, contact Colorado State University Institutional Review Board at [CSU_IRB@colostate.edu](mailto:CSU_IRB@colostate.edu). We will give you a copy of this consent form to take with you.

In case of an emergency or injury due to the study, please contact Colorado State University Institutional Review Board at [CSU_IRB@colostate.edu](mailto:CSU_IRB@colostate.edu) or (970) 491-5241.

Do you give permission for the researchers to contact you again in the future to follow-up on this study or to participate in new research projects? If you indicate yes, your contact details will be saved on a password-protected database on a password-protected computer that only the study’s principal investigators will be able to access.

Please initial next to your choice below.

- Yes, you may retain my contact information and contact me in the future ______ (initials)
- No, you may not retain my contact information nor contact me in the future ______ (initials)

Your signature acknowledges that you have read the information stated and willingly sign this consent form. Your signature also acknowledges that you have received, on the date signed, a copy of this document containing five pages.

PARTICIPANT SIGNATURE (This represents consent from adult participants)

*______________________________________________ _____________________*

Signature of person agreeing to take part in the study Date

______________________________________________

Printed name of person agreeing to take part in the study

_______________________________________________ _____________________

Name of person providing information to participant Date

_____________________________________________

Signature of person providing information to participant.

# fMRI Consent Form

**Consent to Participate in a Research Study**

**Colorado State University**

**Title of Study:** Neural Markers of Static & Dynamic Balance in Adults with Acquired Brain Injury

**Principal Investigators:**

- Jaclyn Stephens, PhD, Dept. of Occupational Therapy

[jaclyn.stephens@colostate.edu](mailto:jaclyn.stephens@colostate.edu)

- Arlene Schmid, PhD, Dept. of Occupational Therapy

[arlene.schmid@colostate.edu](mailto:arlene.schmid@colostate.edu)

- Jennifer Weaver, PhD, Dept. of Occupational Therapy

[jen.weaver@colostate.edu](mailto:jen.weaver@colostate.edu)

- Christopher Bell, PhD, Dept. of Health and Human Sciences

[christopher.bell@colostate.edu](mailto:christopher.bell@colostate.edu)

**What if I have questions?**

Please ask a research team member about any questions you have about this consent or study. For general questions or concerns about the study, you may contact Dr. Jaclyn Stephens at [jaclyn.stephens@colostate.edu](mailto:jaclyn.stephens@colostate.edu). For questions regarding the rights of research subjects, any complaints, or comments regarding the manner in which the study is being conducted, contact the CSU Institutional Review Board at: [CSU_IRB@colostate.edu](mailto:CSU_IRB@colostate.edu) or (970) 491-5241.

**What is the purpose of this study?**

To evaluate functional brain connectivity and task-based brain activity in adults with a history of acquired brain injury who are completing either a group yoga or group exercise intervention.

**Why am I being invited to take part in this research?**

We have invited you to participate in this study because you have sustained an acquired brain injury and demonstrated interest in participating in our study.

**Who is doing the study?**
This study is being conducted by a team of investigators at Colorado State University in the Departments of Occupational Therapy and Health and Exercise Science.

**Where is the study going to take place and how long will it last?**

This portion of the study will take place at Colorado State University Translational Medicine Institute. We will ask you to participate in a pre-intervention and post-intervention assessment. Thus, your participation will be completed over a 10-12 week timeline, which includes the 8-week group yoga or group exercise intervention.

**What will I be asked to do?**

If you agree to participate in this study, you will be asked to complete the following MRI procedures during your pre-intervention and post-intervention assessment visit:

You will be asked to complete an MRI scan. During this session, we will collect Magnetic Resonance images (MRI) to learn about the structure of your brain, using investigational sequences. MRI uses a strong magnetic field and radio waves to take pictures of the brain. You will lay inside the center of a large, doughnut-shaped magnet. Your head will be placed in a special, helmet-like “head coil” which helps us take better pictures. Your scan will be completed by an MRI technologist who will prepare you for the scan, control and monitor the MRI system, and communicate with you throughout the session. You will be asked to lie still and not move. You will hear very loud knocking noises from the machine. We will take several different types of images of your brain, including some of the structure of the brain and some of the function of the brain. During the functional MRI scans, you will be asked to lie still in the scanner and keep your eyes focused on a fixation cross. During task-based portions of the scans, you will be asked to view images and respond to those images. Since several sets of pictures will be collected, this entire procedure will take approximately 45 minutes. Prior to your scan, you will be asked to change into hospital scrubs and to remove all metals from your pockets, rings, watches. etc., which will be securely stored for you. Finally, a research team member will meet with you to describe our research aims in detail, answer any questions that you have about the research, and thank you for your participation.

**Are there reasons why I should not take part in this study?**

Adults who have specific metals or medical devices in their body (e.g., pacemakers) and other conditions that are not compatible with MRI cannot take part in the study. You should not participate if you are claustrophobic. A safety screening form will be used to assess whether it is safe for you to have an MRI.

**What are the possible risks and discomforts?**

It is not possible to identify all potential risks in research procedures, but the research team has taken reasonable safeguards to minimize any known and potential, but unknown, risks. Known risks include:

1. There is a possibility that your personal information could become known outside of the research setting. To prevent this from happening, you will be identified by a randomly assigned ID number and access to identifiable data will be limited to members of the research team
2. MRI uses a large and very strong magnet. Because of this, it may be dangerous for you to enter the scanning room if you have metallic implants, devices, objects, or materials in or on your body. You will be screened before undergoing MRI, and if you have any risk for injury, you will not receive a scan. In addition, all magnetic objects (e.g., cell phones, watches, coins, and jewelry) must be removed before entering the MRI scan room. Individuals with fear of confined spaces may become anxious during MRI. Because the noise from the scanner is very loud, you will be given hearing protection. Please inform the technician if you have sensitive hearing or a hearing disorder (e.g., tinnitus). Although MRI is not painful, and is non-invasive, some people undergoing this procedure experience dizziness, ear ringing, visual spots, or muscle twitches while in or near the scanner. These symptoms are generally mild and last only a short time. There is a possibility of tissue burning if you come in contact with metal during the MRI. For this reason, you should tell the MRI technologist about possible sources on metal in or on your body (e.g., metal buttons, underwire bra, makeup, etc.) so that they can determine whether the procedure is safe for you. Because the scan requires you to lie down and hold still for a long period of time, you might experience back pain or other physical discomforts. MRI may not be safe for human fetuses. Please alert a member of the research team and the MRI technician immediately if you are currently pregnant or could be pregnant (e.g., late menstrual period)
3. While the purpose of this study is for research purposes only, there is an unlikely risk that the scan will reveal a possible brain abnormality. Our research team does not have the clinical background to identify any potential abnormalities. You may choose to share your MRI scan with your doctor. We are not responsible for any medical costs incurred after this point as a result of this potential anomaly.

**Are there any benefits from taking part in this study?**

The benefits that can be expected from this research are related to gaining information about how group yoga or group exercise may help individuals with a history of acquired brain injury. The experiments are not designed to produce any specific health or personal benefits to the subject. This brain scan study is not designed to treat or diagnose any illness. In addition, a copy of the MRI scans can be released to the participants after signing an image release agreement form with the Translational Medicine Institute.

**Do I have to take part in the study?**

Your participation is voluntary. If you decide to participate, you may withdraw your consent and stop participating in any activity, at any time, without penalty or loss of benefits to which you are otherwise entitled.

**What will it cost me to participate?**

There are no costs to you associated with participation in this study.

**Who will see the information that I give?**

We will keep private all research records that identify you, to the extent allowed by law. Your information will be combined with information from other research participants taking part in the study. When we write about the study to share it with other researchers, we will write about the combined information that we have gathered. You will not be identified in these written materials. We may publish the results of this study; however, we will keep your name and other identifying information private. We will make every effort to prevent anyone who is not on the research team from knowing that you gave us information. For example, your name will be kept separate from your research records, and these two things will be stored in different places. You will be given an ID number that will be used for all materials associated with your participation. Exceptions include if we are asked to share the research files for audit purposes with the CSU Institutional Review Board ethics committee. Your identity and record of compensation (NOT your data) may be made available to CSU officials for financial audits. Your data may be shared with researchers outside of the research team, or used for teaching purposes, but only after they have been de-identified (i.e., removed of all identifying information). The MRI collected in this study is for research purposes only. Researchers do not have training to evaluate the individual images for diagnostic purposes. In rare instances, your images might be seen by a licensed medical professional for the purpose of reviewing abnormal findings; however, we cannot guarantee that all abnormal findings will be reviewed by a medical professional. A copy of the data can be released to you at the end of the session.

**Can my taking part in the study end early?**

Your participation in this study may be stopped by the investigators at any time, with or without your consent.

**Will I receive any compensation for taking part in this study?**

You will not receive any additional compensation for taking part in this portion of the study.

**What happens if I am injured because of the research?**

The Colorado Governmental Immunity Act determines and may limit Colorado State University's legal responsibility if an injury happens because of this study. Claims against the University must be filed within 180 days of the injury. Should you need medical assistance, you or your health insurance will be responsible for the costs.

**May we contact you about future MRI research opportunities?**

*Please respond* [    ] YES   [    ] NO

**Signed Consent**

Your signature acknowledges that you have read the information stated and willingly sign this consent form. Your signature also acknowledges that you have received, on the date signed, a copy of this document containing four pages.

_________________________________________ __________

Signature of person agreeing to take part in the study   Date

____________________________________________

Printed name of person agreeing to take part in the study

___________________________________ __________

Signature of researcher obtaining consent   Date

_____________________________________

Printed name of researcher obtaining consent
